# Inflammatory myofibroblastic tumor in the larynx

**DOI:** 10.1016/S1808-8694(15)30619-4

**Published:** 2015-10-18

**Authors:** Daniel Matos Barreto, Luciano Freitas Rodrigues, Lucas Gomes Patrocinio, Sonia Regina Coelho, José Antonio Patrocinio

**Affiliations:** 1 MD, resident at the otorhinolaryngology service at Faculdade de Medicina da Universidade Federal de Uberlândia.; 2 MD, resident at the otorhinolaryngology service at Faculdade de Medicina da Universidade Federal de Uberlândia.; 3 ENT, physician at the otorhinolaryngology service at Faculdade de Medicina da Universidade Federal de Uberlândia.; 4 MSc, Head of the Laryngology and Voice Division at the Otorhinolaryngology service at Faculdade de Medicina da Universidade Federal de Uberlândia.; 5 Professor, Head of the Otorhinolaryngology service at Faculdade de Medicina da Universidade Federal de Uberlândia. Department of Otorhinolaryngology, Hospital Santa Genoveva, Uberlândia, Minas Gerais, Brazil.

**Keywords:** squamous cell carcinoma, differential diagnosis, laryngeal neoplasms

## INTRODUCTION

Inflammatory myofibroblastic pseudotumors were initially described in the lung, only then to be identified in various extrapulmonary sites^1^. In the head and neck, this disease is more frequently found in the paranasal sinuses, and has already been described in the orbits, palatine tonsils, ears, gingiva, pterygomaxillary space, and periodontal tissues. It will rarely involve the larynx. Only twenty-two cases of inflammatory myofibroblastic pseudotumor in the larynx have been described in the indexed literature^2^, ^3^, ^4^, ^5^.

This paper aims to describe a rare case of laryngeal inflammatory myofibroblastic pseudotumor and discuss its diagnostic and therapeutic aspects.

## CASE REPORT

J.G., male, 22 years-old, complained about having persistent hoarseness for two months. He claimed not to smoke, abuse his voice, or use medication, and had no gastroesophageal complaints or allergies. Videolaryngoscopy revealed redness and edema on the right vocal fold and a polypoid lesion (Picture 1A). The patient was referred to laryngeal microsurgery to have the lesion removed. Pathology tests indicated the polypoid lesion was covered by a non-keratinizing, squamous epithelium mucosa, and a chorion with cellular and fascicular proliferation, made up by fusiform and star-shaped cells resembling fibroblasts or myofibroblasts, rarely with a histiocytic aspect, with prominent chronic inflammatory infiltrate rich in plasmacytes and polymorph nuclei (Picture 1B). Immunohistochemistry showed diffuse positive results for vimentin and focal positive results for 1A4 smooth muscle actin, and was negative for cytokeratins (AE1AE3) and desmin. Final diagnosis was inflammatory myofibroblastic pseudotumor.

**Picture 1:**
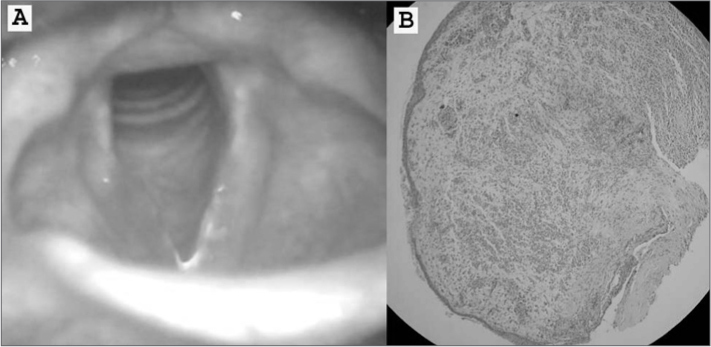
(A) Videolaryngoscopy revealing redness and right vocal fold edema; (B) polypoid lesion covered by non-keratinizing, squamous epithelium, with a chorion with fusiform cells resembling fibroblasts or myofibroblasts (hematoxylin-eosin, 200X).

After surgery the patient reported improvements in his voice, and had a normal videolaryngoscopy 30 days after surgery. He came back six months after surgery complaining of hoarseness again. Videolaryngoscopy revealed a relapsing polypoid lesion. Another surgical procedure was carried out and the polyp was removed without expanding the surgical margins. Pathology tests confirmed the presence of inflammatory myofibroblastic pseudotumor. The patient has not had any voice-related complaints for 12 months, and his videolaryngoscopy has been normal ever since.

## DISCUSSION

Laryngeal inflammatory myofibroblastic pseudotumors represent a rare disease. It was first described in 1992, and is possibly a challenging condition from the diagnostic point of view^2^. The lesion looks clinically suspicious and, histologically, it mimics squamous cell carcinomas^3^. The nature of this disease is still uncertain.

Inflammatory myofibroblastic pseudotumors were initially considered to be a non-tumor lesion, being possibly associated with abnormal inflammatory response^2^. Today, this condition is seen as a neoplasm due to the following characteristics: potential for relapsing locally, development of non-adjacent multifocal tumors, infiltrative local growth, vascular invasion, and even distal metastasis. Histologically speaking, the lesions are quite similar to malignant tumors (squamous cell carcinoma, leiomyosarcoma, malignant histiocytoma), and require complementary immunohistochemistry tests^4^, ^6^.

Laryngeal inflammatory myofibroblastic pseudotumors are extremely rare, with only 22 cases described in the literature^2^, ^3^, ^4^, ^5^. Eighty percent of the patients have this laryngeal disease on their vocal folds. The most common symptoms are apparently hoarseness and dysphonia. Under videolaryngoscopy the lesion is often pedunculated, with a polypoid aspect, but may also present itself as a nodular elevation. Size varies between 0.4cm and 3.5cm. Prognosis is yet unclear, but appears to be favorable as few are the cases of local relapse and complications reported^4^, ^5^.

## CONCLUSION

Inflammatory myofibroblastic pseudotumors must be included in the differential diagnosis of malignant laryngeal tumors, both due to its reduced aggressiveness and the possibility of treating it through conservative surgery, unlike neoplasms. Further studies are required to determine the true nature and evolution of this laryngeal infirmity.
